# Empowering Materials Processing and Performance from Data and AI

**DOI:** 10.3390/ma14164409

**Published:** 2021-08-06

**Authors:** Francisco Chinesta, Elias Cueto, Benjamin Klusemann

**Affiliations:** 1 PIMM Laboratory & ESI Group Chair, Arts et Métiers Institute of Technology, CNRS, Cnam, HESAM Université, 151 Boulevard de l’Hôpital, 75013 Paris, France; francisco.chinesta@ensam.eu; 2 ESI Group, Bâtiment Seville, 3bis Rue Saarinen, 50468 Rungis, France; 3 Aragon Institute of Engineering Research, Universidad de Zaragoza, 50009 Zaragoza, Spain; ecueto@unizar.es; 4 Institute of Product and Process Innovation, Leuphana University of Lüneburg, 21335 Lüneburg, Germany; 5 Institute of Materials Mechanics, Helmholtz-Zentrum Hereon, 21502 Geesthacht, Germany

Third millennium engineering is addressing new challenges in materials sciences and engineering. In particular, the advances in materials engineering, combined with the advances in data acquisition, processing and mining as well as artificial intelligence, allow new ways of thinking in designing new materials and products. Additionally, this gives rise to new paradigms in bridging raw material data and processing to the induced properties and performance. On the one hand, the linkage can be done purely on a data-driven basis, i.e., models are created from scratch based on the obtained experimental data alone, for instance with statistical methods or advanced methods of machine learning. Particularly obvious advantages of such kinds of models are that no simplification or assumptions need to be incorporated a priori, and that it allows real-time prediction, leading to a so-called digital twin of the specific material/process. However, such approaches typically face some general challenges, such as the necessity of (maybe unnecessarily) large and comprehensive datasets, because they rely only on the data themselves and allow prediction only within the investigated/trained dataspace. Another way of addressing the challenge of predicting the complex processing–structure–property relationships in materials is the enhancement of already existing physics-based models via data and machine learning tools, i.e., combining a physics-based model (often called virtual twin) and a data-based model, leading to a so-called hybrid twin [1]. In this regard, possible deviations of the physics-based model, which rely on a number of simplifications and assumptions, can be healed by correcting the model based on a data-driven approach, i.e., combining the advantages of both models. 

This present topical issue is a compilation of contributions on novel ideas and concepts, addressing several key challenges using data and artificial intelligence:

- proposing new techniques for data generation and data mining;
- proposing new techniques for visualizing, classifying, modeling, extracting knowledge, explaining and certifying, data and data-driven models;
- processing data, for creating data-driven models from scratch when other models are absent, too complex or too poor for making valuable predictions;
- processing data for enhancing existing physic-based models to improve the quality of the prediction capabilities, and at the same time enabling data to be smarter.

Gong et al. [2] explored the material behavior of additive Ti-Mn samples, produced via the Laser Engineered Net Shaping (LENS™) technique and subsequent post-build heat treatment. In this work, novel cost-effective high-throughput experimental assays have been proposed and the feasibility of employing Gaussian process regression (GPR) for in-depth statistical analysis of these data has been shown. From the additive-manufactured compositionally graded cylinders and after heat treatment, samples were extracted for microstructural analysis and the mechanical properties were systematically determined via spherical indentation. The proposed approach allows to explore large material and process spaces. Via GPR, the process–microstructure–properties relationships were analyzed. The statistical approach showed a reliable and robust correlation between the Mn content and the specific ageing conditions to the microstructural parameters for the obtained data via the proposed high-throughput assays. 

Huber [3] analyzed the microstructure–property relationships in nanoporous metals. In this study, a clear recommendation is given to always start with a dimensional analysis, ensuring a physically reasonable formulation of the problem at hand and a reduction of the problem to its minimum This included the incorporation of already existing physical knowledge by turning inputs and outputs into dimensionless parameters via physical normalization. The nanoporous microstructure was analyzed based on datasets obtained from finite element calculations of a beam model of the structure, investigated for macroscopic compression and nanoindentation. To analyze the microstructure–property relationships, not only dimensional analysis but also principal component analysis and visualization are used on the obtained datasets, where, in particular, machine learning algorithms are employed to identify key dependencies that allowed for deriving simple linear relationships in this case. It is revealed that the “scaling law of the work hardening rate has the same exponent as the Young’s modulus” [3], an insight which can be transferred to the macroscopic behavior of such structures, allowing designing and tuning of such microstructures in terms of the specific objective. 

Yun et al. [4] address the construction of models based on data involving complex microstructures. When addressing these complex microstructures, concise and complete morphological and topological descriptors are needed, as well as adequate metrics able to quantify the resemblance and proximity of different microstructures for which standard Euclidian distances do not perform correctly. For that purpose, Topological Data Analysis (TDA) is considered, and thus microstructures are represented from the so-called persistence images that contain most of the microstructural information while inheriting the appealing invariance topological features. With the persistence images defined in a vector space, usual metrics can be used to apply machine learning and data analytics techniques. Thus, the persistence images can be considered as inputs in machine learning-based nonlinear regressions, to infer the properties and performances associated with those complex microstructures. In particular, this work addresses the prediction of the effective thermal conductivity, inferred from the persistence image, without the need of further calculations.

For the identification and calibration of complex constitutive models, de Pablos et al. [5] presented an effective framework consisting of a two-stage procedure. In the first step, a metamodel is formulated and used to perform a Global Sensitivity Analysis, providing the sensitivity of each parameter in the selected material model with respect to the Quantities of Interest. Anisotropic Radial Basis Functions are used as kernel functions to build the linear metamodels in this work, allowing the generation of a large number of data points. The second step represent the parameter calibration for the most influential parameters based on Bayesian inference in combination with the application of Gaussian processes. This calibration process leads not only to the optimal mean value of the parameters, but it also provides information about their probability distributions. In this regard, the framework can help to select necessary subsequent experiments, but at the same time minimize the required number of experiments and numerical calculations. The application of the framework is illustrated on three well-known constitutive laws to describe elasto-viscoplastic material behavior.

Hartmaier [6] derived a novel mathematical formulation for a data-oriented constitutive model for elastic–plastic materials. In particular, this model consists of the identification of the yield function via a support vector characterization (SVC) algorithm to describe the elastic–plastic deformation. The model can be easily incorporated within finite element calculations. To reduce the dimensionality of the problem and without loss of generality, core physical principles are incorporated, i.e., working with the deviatoric stress as input data. The SVC algorithm can easily be trained, for instance, only based on the information whether a given stress state is within the elastic regime or not, leading to a machine learning yield function from which further information, such as its gradient, can be easily calculated. Overall, the presented data-oriented constitutive model shows a great flexibility in terms of application to all kind of anisotropy for elastic–plastic materials, where, for instance, the standard frameworks of continuum plasticity can still be employed within a finite element set-up. Identifying macroscopic yield functions based on microscopic calculations, the presented formulation is directly applicable as a kind of scale-bridging approach. 

For scale bridging, Lu et al. [7] proposed a stochastic data-driven multilevel finite element (FE2) method, illustrated by the example of nonlinear electric conduction in graphene–polymer composites. At the microlevel, finite-element calculations of representative volume elements (RVE) of the graphene-polymer composites serve for the purpose of data generation. The data are used to train a neural network surrogate model, which relates the macroscopic electric field and volume fraction of graphene inclusions to the macroscopic electric flux. To reduce the necessary number of RVE calculations and at the same time to improve the accuracy of the surrogate model, a novel hybrid neural network–interpolation scheme is proposed. Since the surrogate model is computationally extremely cheap, it can efficiently be employed in a multilevel finite element approach. Additionally, considering certain properties as a stochastic field with given probabilistics at the macroscale, e.g., in the current study the volume fraction of heterogeneities, the surrogate model is used to identify a probabilistic model for each point at the macroscale, leading to a data-driven approach in a stochastic framework. Due to the computational efficiency, Monte Carlo simulations can be performed, allowing this approach to account for uncertainties at both scales. 

Gonzalez et al. [8] deal with a particularly frequent problem in biomechanics: the large deviation of experimental results. In this framework, the standard deviation of the measurements may take a similar value to that of their mean value, thus making regression procedures a delicate issue. When this circumstance is present, they propose a combination of TDA and regression procedures operating on these just found data manifolds. To ensure the compliance to basic thermodynamic principles (conservation of energy, production of entropy), they suggest performing regression over a particularly interesting principle: the so-called General Equation for Non-Equilibrium Reversible-Irreversible Coupling (GENERIC). Generic is in fact a metriplectic formalism that, subject to adequate degeneracy conditions, satisfies by construction the first and second principles of thermodynamics. The resulting method thus ensures the thermodynamic correctness of the obtained constitutive laws, while dealing with the just-mentioned large deviations in experimental data.

Bock et al. [9] present a hybrid twin model for the use case of laser shock peening. The hybrid model consists of a physics-based model, i.e., a semi-analytical model, which is enhanced by a trained artificial neural network. The latter is accounting for the present deviation to a reference (high-fidelity) solution, which is determined by a finite element simulation of the laser shock peening process. On the one hand, the hybrid twin allows for a more efficient prediction of the residual stress field within the material in comparison to the high-fidelity model, since the computational costs are significantly reduced and similar to the ones required by the semi-analytical model. On the other hand, the hybrid twin outperforms a purely data-driven model by exhibiting enhanced generalization capabilities, i.e., predictions outside the process parameter region used for training, while requiring less data than the data-driven approach. The importance of a dimensionality analysis to identify the salient input features is emphasized, leading to low prediction errors. 

Overall, the original articles compiled in this Research Topic give a taste of current ideas and concepts in how to enhance existing knowledge via new concepts of artificial intelligence to address current challenges in materials sciences and engineering. Such approaches provide new pathways, in particular in the research field of computational engineering, but with high impact on different engineering fields, such as material science and processing. At this point, as guest editors, we would like to thank all authors for their valuable contributions to this Special Issue!
